# Supplementary material for: The Chimeric Peptide (GEP44) Reduces Body Weight and Both Energy Intake and Energy Expenditure in Diet-Induced Obese Rats
Source: Int J Mol Sci. 2025 Mar 26;26(7):3032. doi: 10.3390/ijms26073032 (PMC11989200; doi:10.3390/ijms26073032)
Supplement: Supplementary file 1 [file ijms-26-03032-s001.zip › ijms-3482497-supplementary.pdf]

Supplemental Table S1. Range of detectability for plasma hormones

Plasma hormone	Range of Detectability
Adiponectin	0.25-10 ng/mL
FGF-21	0.0313-2 ng/mL
Glucagon	6.97-633.9 pg/ml
Insulin	0.069-50 ng/mL
Irisin	78-5,000 ng/mL
Leptin	0.07-51.9 ng/mL

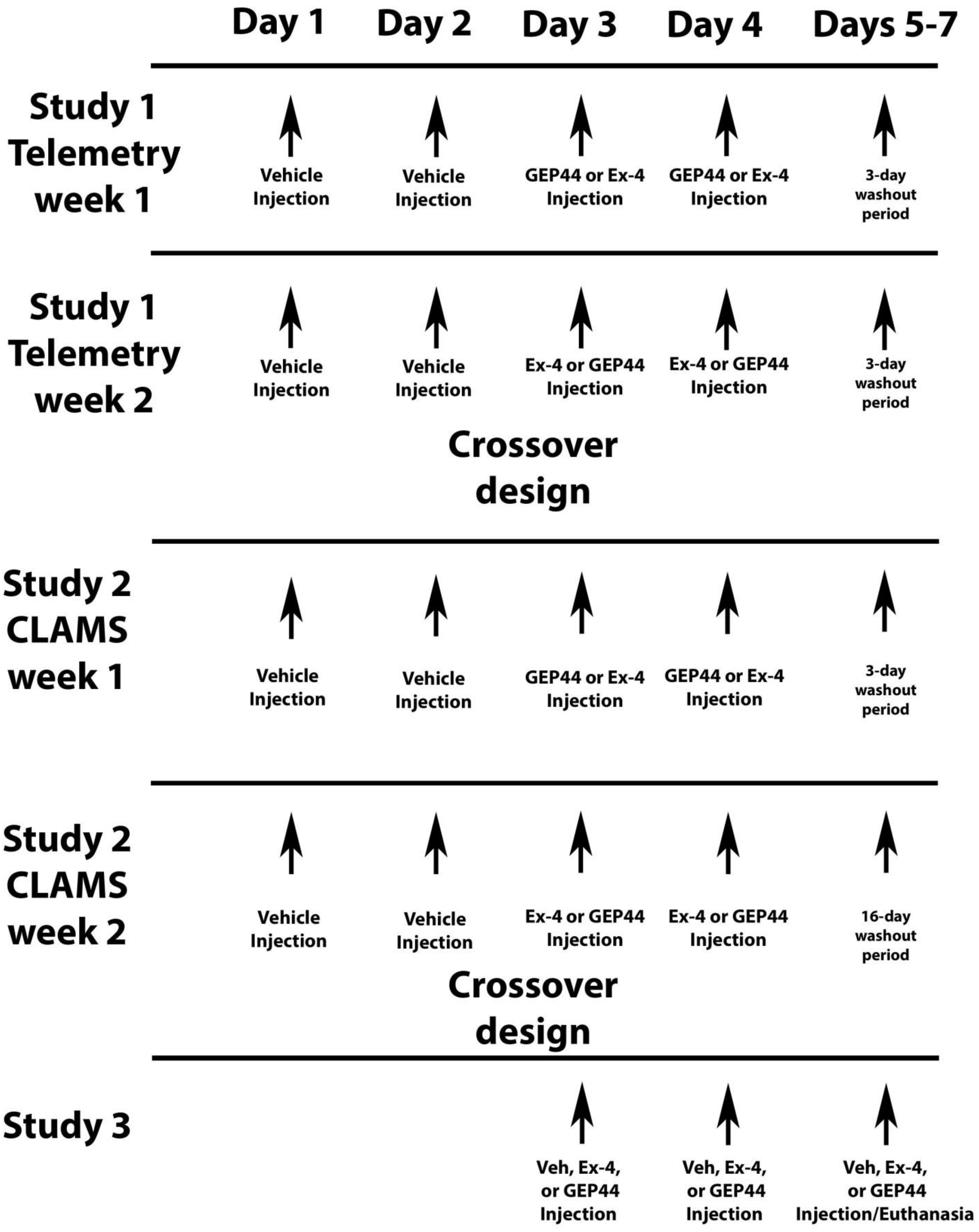

Supplemental Figure S1: Schematic of Experimental Paradigms Used in Studies 1, 2 and 3.

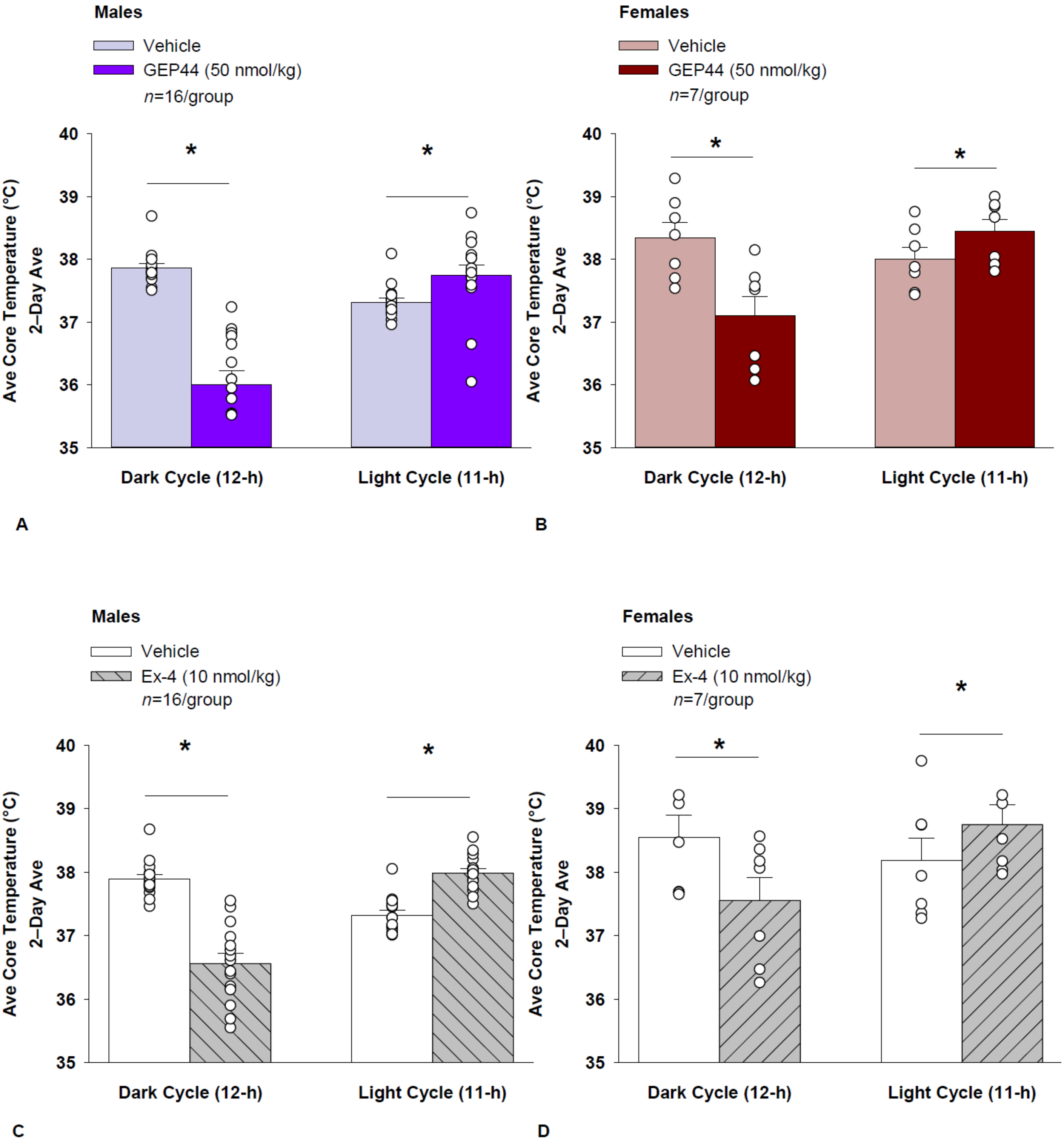

Supplemental Figure S2A–D: Effects of the chimeric peptide, GEP44, or selective GLP-1R agonist, exendin-4, on core temperature in male and female HFD-fed rats. Male (*n*=15/group) and female (*n*=7/group) rats were maintained on HFD (60% kcal from fat) for at least 4 months prior to being implanted with PDT-4000 telemetry devices into the abdominal cavity. During this study, animals remained in their home cages and subsequently received SC injections of vehicle (sterile saline/water) followed by GEP44 (50 nmol/kg; 1 mL/kg injection volume) or exendin-4 within 15 min prior to the start of the dark cycle in a counterbalanced design. Core temperature data was collected using the PDT-4000 telemetry devices. A-B, Effect of GEP44 on core temperature during the light and dark cycle periods in A) male and B) female HFD fed rats; C-D, Effect of exendin-4 on core temperature during the light and dark cycle periods in C) male and D) female HFD-fed rats. Core temperature was averaged at 11-h light and 12-h dark cycle periods over the 2-day vehicle and 2-day drug treatment period. Data are expressed as mean ± SEM. *$p<0.05$ GEP44 or exendin-4 vs. vehicle.

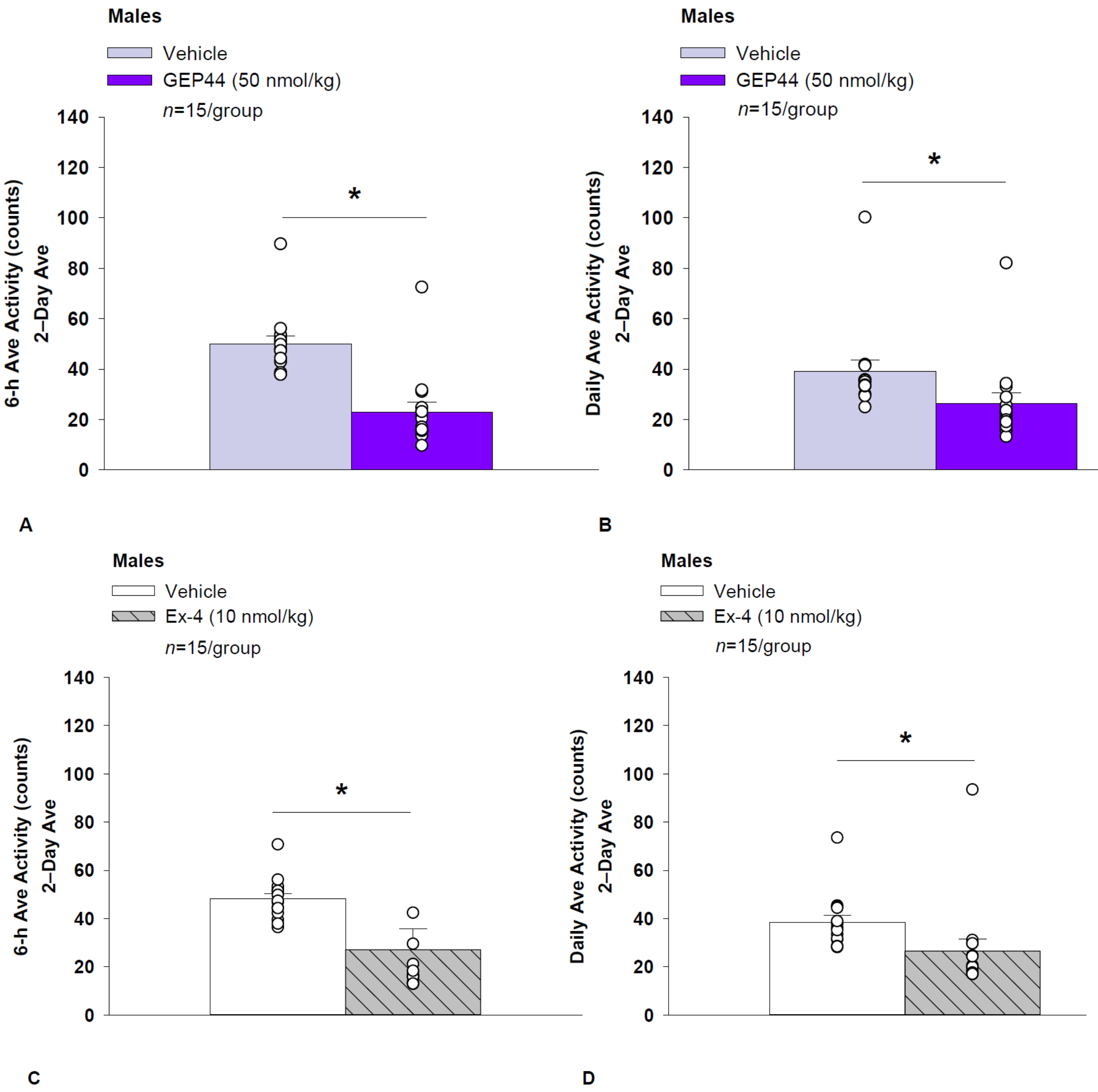

Supplemental Figure S3A–D: Effects of the chimeric peptide, GEP44, or selective GLP-1R agonist, exendin-4, on gross motor activity over 6-h post-injection in male HFD-fed rats. Male (*n*=15/group) rats were maintained on HFD (60% kcal from fat) for at least 4 months prior to being implanted with PDT-4000 telemetry devices into the abdominal cavity. During this study, animals remained in their home cages and subsequently received SC injections of vehicle (sterile saline/water) followed by GEP44 (50 nmol/kg; 1 mL/kg injection volume) or exendin-4 within 15 min prior to the start of the dark cycle in a counterbalanced design. Gross motor activity data was collected using the PDT-4000 telemetry devices. A, Effect of GEP44 on 6-h activity in male HFD-fed rats; B, Effect of GEP44-4 on daily (23-h) activity in male HFD-fed rats; C, Effect of exendin-4 on 6-h activity in male HFD-fed rats; D, Effect of exendin-4 on daily (23-h) activity in male female HFD-fed rats. Activity was averaged at 6-h and 23-h post-treatment periods over the 2-day vehicle and 2-day drug treatment period. Data are expressed as mean ± SEM. *$p<0.05$ GEP44 or exendin-4 vs. vehicle.

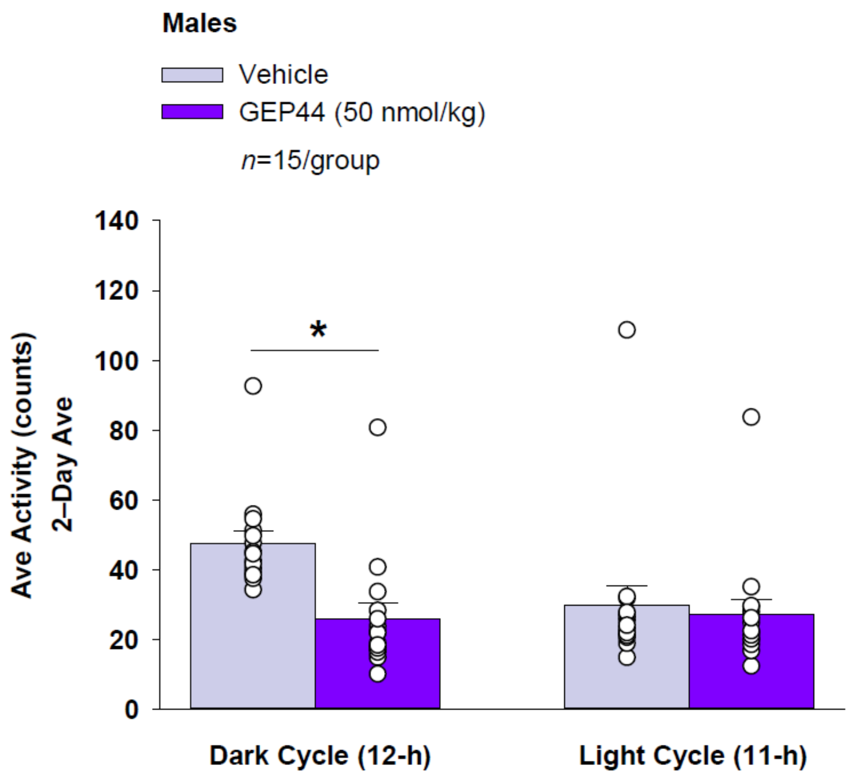

A

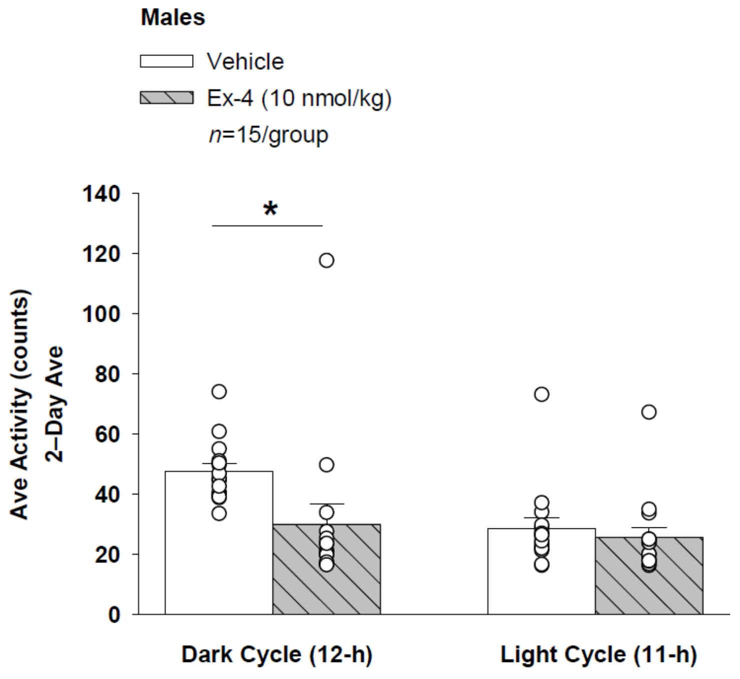

B

Supplemental Figure S4A–B: Effects of the chimeric peptide, GEP44, or selective GLP-1R agonist, exendin-4, on gross motor activity over the light and dark cycles in male HFD-fed rats. Male (*n*=15/group) rats were maintained on HFD (60% kcal from fat) for at least 4 months prior to being implanted with PDT-4000 telemetry devices into the abdominal cavity. During this study, animals remained in their home cages and subsequently received SC injections of vehicle (sterile saline/water) followed by GEP44 (50 nmol/kg; 1 mL/kg injection volume) or exendin-4 within 15 min prior to the start of the dark cycle in a counterbalanced design. Gross motor activity data was collected using the PDT-4000 telemetry devices. *A*, Effect of GEP44 on activity during the light and dark cycle periods in male HFD-fed rats; *B*, Effect of exendin-4 on activity during the light and dark cycle periods in male HFD-fed rats. Activity was averaged at 11-h light and 12-h dark cycle periods over the 2-day vehicle and 2-day drug treatment period. Data are expressed as mean ± SEM. *p<0.05 GEP44 or exendin-4 vs. vehicle.
